# Curricula for teaching the content of clinical practice guidelines to family medicine and internal medicine residents in the US: a survey study

**DOI:** 10.1186/1748-5908-4-59

**Published:** 2009-09-21

**Authors:** Elie A Akl, Reem Mustafa, Mark C Wilson, Andrew Symons, Amir Moheet, Thomas Rosenthal, Gordon H Guyatt, Holger J Schünemann

**Affiliations:** 1 Department of Medicine, State University of New York at Buffalo, NY, USA; 2 Department of Family Medicine, State University of New York at Buffalo, NY, USA; 3 Department of Internal medicine, University of Iowa, IA, USA; 4 Department of Medicine, Rochester General Hospital, Rochester, NY, USA; 5 Department of Medicine, University of Rochester, NY, USA; 6 Department of Medicine, McMaster University, Hamilton, ON, Canada; 7 Department of Clinical Epidemiology & Biostatistics, CLARITY Research Group, McMaster University, Hamilton, ON, Canada

## Abstract

**Background:**

Teaching the content of clinical practice guidelines (CPGs) is important to both clinical care and graduate medical education. The objective of this study was to determine the characteristics of curricula for teaching the content of CPGs in family medicine and internal medicine residency programs in the United States.

**Methods:**

We surveyed the directors of family medicine and internal medicine residency programs in the United States. The questionnaire included questions about the characteristics of the teaching of CPGs: goals and objectives, educational activities, evaluation, aspects of CPGs that the program teaches, the methods of making texts of CPGs available to residents, and the major barriers to teaching CPGs.

**Results:**

Of 434 programs responding (out of 839, 52%), 14% percent reported having written goals and objectives related to teaching CPGs. The most frequently taught aspect was the content of specific CPGs (76%). The top two educational strategies used were didactic sessions (76%) and journal clubs (64%). Auditing for adherence by residents was the primary evaluation strategy (44%), although 36% of program directors conducted no evaluation. Programs made texts of CPGs available to residents most commonly in the form of paper copies (54%) while the most important barrier was time constraints on faculty (56%).

**Conclusion:**

Residency programs teach different aspects of CPGs to varying degrees, and the majority uses educational strategies not supported by research evidence.

## Background

The implementation of clinical practice guidelines (CPGs) has been shown to positively impact clinical care. For example, the implementation of community-acquired pneumonia guidelines has decreased mortality [1] and improved cost effectiveness of care [2] without adverse patient consequences [3]. Diabetes care employing evidence-based guidelines [4] reduced diabetic nephropathy, retinopathy, and autonomic neuropathy. Improved survival in women with node-negative breast cancer has been associated with compliance with guidelines for systemic adjuvant treatment [5]. Use of guidelines for the management of presumed uncomplicated urinary tract infection in women has decreased laboratory utilization and overall costs while maintaining or improving the quality of care [6].

On the post graduate medical education level, teaching the content of CPGs can help in achieving three of the six general competencies defined by the Accreditation Council for Graduate Medical Education (ACGME) in the United States (US) [7]: patient care, medical knowledge, and practice-based learning and improvement [8]. It is also assumed that such teaching will help trainees to adhere to CPGs when they become independent practitioners.

In spite of their clinical and educational values, the implementation of CPGs remains suboptimal among family medicine and internal medicine trainees in the US for a number of clinical areas such as: management of hypertension [9]; management of hypercholesterolemia [10]; diagnosis and management of uncomplicated urinary tract infection [11]; screening and management of chronic kidney disease and its associated co-morbidities [12]; and screening for familial colorectal cancer [13].

One of the potential explanations for the suboptimal implementation of CPGs is suboptimal teaching of CPGs in residency programs. The main objective of this study was to determine the characteristics of curricula for teaching the content of CPGs in family medicine and internal medicine residency programs in the US. We also wanted to explore the hypothesis that a lower percentage of international medical graduates would be associated with better characteristics of teaching CPGs. It was not our objective to study either informal teaching (*i.e.*, outside the curriculum, *e.g.*, bedside teaching) or teaching of guidelines' development or guidelines' integration in medical decision making.

## Methods

### Study design

We conducted a national survey of directors of family medicine and internal medicine residency programs in the US. We identified the program directors and obtained their contact information using the American Medical Association Graduate Medical Education Directory [14]. The University at Buffalo Institutional Review Board approved the study.

### Survey questionnaire

To develop the questionnaire, we reviewed the medical literature including the ACGME definition of a curriculum [15] and the Cochrane Effective Practice and Organisation of Care (EPOC) review group typology of implementation interventions [16]. We also conducted discussions with five internal medicine chief residents and two program directors attending the American College of Physicians (ACP) and the Association of Program Directors in Internal Medicine (APDIM) 2005 annual meetings. Two current and one previous program director reviewed iterative versions of the questionnaire and provided feedback.

The survey questionnaire included questions about the characteristics of the teaching of CPGs: goals and objectives, educational activities, evaluation, aspects of CPGs that the program teaches, the methods of making texts of CPGs available to residents, and the major barriers to teaching CPGs. The questionnaire included additional questions about the characteristics of the program director (gender, years as program director) and the characteristics of the residency program (geographical region, affiliation, number of residents, and percentage of international medical graduates) (Additional File 1).

### Data collection

We mailed program directors the initial invitation to participate in the survey in April 2007. We used the following survey methods demonstrated to maximize response rate [17,18]: university sponsorship, personalized cover letter, colored ink, stamped return envelope, first class mailing, follow-up mail, including a questionnaire in the follow-up mail, non-monetary incentive, and a questionnaire that is interesting, short, user friendly, with factual questions, and with more relevant questions first. The non-monetary incentive consisted of a Microsoft PowerPoint version of an educational game designed to teach CPGs and using rules similar to those of the TV show Jeopardy^® ^[19]. We sent a follow-up mail and a follow-up fax respectively five and nine weeks after the initial invitation.

### Statistical analysis

We conducted the descriptive analyses for the two specialties (family medicine and internal medicine) separately and combined. We conducted regression analyses in order to identify factors that are associated with the characteristics of the teaching of CPGs. We used logistic models with each of the options for the characteristics of the teaching of CPGs as a dependent variable, and the specialty, the program director characteristics, and the residency program characteristics as the independent variables (reference categories for the categorical variables were respectively: internal medicine specialty, male gender, Northeast geographical region, community based programs, and <25% international medical graduates). We report only statistically significant associations with odds ratio (OR) <0.8 or OR <1.25. We used Microsoft Office Access for data entry and management and SPSS, version 13.0 (SPSS, Inc., Chicago, Illinois), for all analyses.

## Results

The survey overall response rate was 52% (434 out of 839; 52% and 51% of family medicine and of internal medicine program directors responding, respectively). Table 1 lists the characteristics of the training programs combined and separately for the two specialties.

**Table 1:** The characteristics of responding program directors and of their residency programs; a national survey, 2007

	**Combined**	**Family Medicine**	**Internal Medicine**
	**N = 434**	**N = 239**	**N = 195**

**Gender of program director**, n (%)			

Female	98 (23)	48 (20)	50 (26)

**Years as director**, mean (SD)	7.3 (5.4)	6.9 (5.1)	7.7 (5.6)

**Geographical region**, n (%)			

Northeast	129 (30)	47 (20)	82 (42)

South	112 (26)	69 (29)	43 (22)

Midwest	119 (27)	79 (33)	40 (21)

West	68 (16)	40 (17)	28 (14)

**Affiliation**, n (%)			

Community based	294 (68)	182 (76)	112 (57)

University based	113 (26)	41 (17)	72 (37)

Military based	15 (3)	8 (3)	7 (4)

Other	6 (1)	4 (2)	2 (1)

**Number of residents per program**, mean (SD)	36.5 (28.5)	22.0 (8.1)	53.8 (34.0)

**International graduates**, n (%)			

<25%	174 (40)	108 (43)	66 (34)

25 to 50%	85 (20)	53 (22)	32 (16)

51 to 75%	76 (18)	43 (18)	33 (17)

>75%	94 (22)	31 (13)	63 (32)

Table 2 presents the answers of responding program directors relating to questions about teaching of CPGs combined and separately for the two specialties.

**Table 2:** Answers to questions relating to teaching of the content of clinical practice guidelines; a national survey, 2007

	**Combined**	**Family Medicine**	**Internal Medicine**
	**N = 434**	**N = 239**	**N = 195**

**Goals and objectives**			

Yes	61 (14)	32 (13)	29 (15)

**Aspects of CPGs taught**			

Identifying and locating CPGs	310 (71)	191 (80)	119 (61)

Critical appraisal of CPGs	222 (51)	130 (54)	92 (47)

Content of specific CPGs	329 (76)	185 (77)	144 (74)

Dealing with conflicting CPGs	127 (29)	82 (34)	45 (23)

None	37 (9)	12 (5)	25 (13)

Other	20 (4.6)	15 (6.2)	5 (2.6)

**Educational activities to teach CPGs**			

Making texts of CPGs available	229 (53)	126 (53)	103 (53)

Didactic sessions	331 (76)	196 (82)	135 (69)

Interactive sessions	154 (36)	83 (35)	71 (36)

Journal club	276 (64)	157 (67)	119 (61)

Audit and feedback to residents *	163 (38)	99 (41)	64 (33)

Self-audit by residents	90 (21)	50 (21)	40 (21)

Educational games	75 (17)	41 (17)	34 (17)

None	23 (5)	5 (2)	18 (9)

Other	25(5.8)	15 (6.3)	10 (5.1)

**Evaluation of the teaching of CPGs**			

Objective assessment of knowledge	151 (35)	79 (33)	72 (37)

Assessment of attitude	45 (10)	23 (10)	22 (11)

Auditing of resident adherence *	190 (44)	123 (51)	67 (34)

Assessment of satisfaction	40 (9)	23 (10)	17 (9)

None	157 (36)	78 (33)	79 (41)

Other	17 (3.9)	9 (3.7)	8 (4.1)

**CPGs texts made available through**			

The website of the program	142 (33)	79 (33)	63 (32)

Servers of affiliated hospital(s)	181 (42)	102 (43)	79 (41)

email distribution	76 (18)	44 (18)	32 (16)

Personal digital assistant (PDA)	107 (25)	85 (36)	22 (11)

Paper copies	236 (54)	136 (57)	100 (51)

None	20 (5)	7 (3)	13 (7)

Other	47 (10.8)	25 (10.46)	22 (11.3)

**Barriers**			

Limited access to CPGs	31 (7)	13 (5)	18 (9)

Insufficient interest among residents	72 (17)	35 (15)	37 (19)

Insufficient interest among faculty	96 (22)	41 (17)	55 (28)

Time constraints on residents	214 (49)	118 (49)	96 (49)

Time constraints on faculty	245 (56)	140 (59)	105 (54)

None	94 (22)	53 (22)	41 (21)
	35 (8.0)	22 (9.2)	13 (6.7)

*Audit can be used as an evaluation strategy but also as an educational strategy if coupled with by feedback or conducted by the residents themselves.

CPGs = Clinical Practice Guidelines

### Written goals and objectives

Fourteen percent of responding program directors reported having written goals and objectives for teaching CPGs.

### Aspects of CPGs

The most frequently taught aspect of CPGs was the content of specific CPGs (76%). Nine percent of program directors reported teaching no aspect of CPGs. Teaching how to identify and locate CPGs and teaching critical appraisal of CPGs were associated with family medicine specialty (OR = 2.70; 95% Confidence Interval (CI) 1.52 to 4.80) and OR = 1.81; 95% CI 1.10 to 3.07 respectively). Teaching the content of specific CPGs was inversely associated with 51 to 75% of residents being international medical graduates (OR = 0.44; 95% CI 0.22 to 0.88). Teaching how to deal with conflicting CPGs was associated with female gender of the program director (OR = 2.00; 95% CI 1.17 to 3.38), and inversely associated with 26 to 50% of residents being international medical graduates (OR = 0.45; 95% CI 0.23 to 0.89).

### Educational activities

The top educational activity was didactic sessions (76%). Five percent of program directors reported using no educational activity.

Didactic sessions were associated with family medicine specialty (OR = 2.27; 95% CI 1.24 to 4.15). Journal clubs was inversely associated with 51 to 75% of residents being international medical graduates (OR = 2.05; 95% CI 1.07 to 3.92). Audit and feedback was inversely associated with 26 to 50% of residents being international medical graduates (OR = 0.49; 95% CI 0.27 to 0.49). Interactive sessions were associated with 26 to 50% of residents being international medical graduates (OR = 1.78; 95% CI 1.02 to 3.08).

### Evaluation

The most frequently reported evaluation strategy was auditing of residents' adherence (44%). Thirty six percent of program directors reported conducting no evaluation.

Auditing adherence to CPGs was associated with family medicine specialty (OR = 1.72; 95% CI 1.01 to 2.94) and inversely associated with 26 to 50% of residents being international medical graduates (OR = 0.45; 95% CI 0.26 to 0.80). Conducting objective assessment of knowledge was inversely associated with family medicine specialty (OR = 0.52; 95% CI 0.30 to 0.90).

### Making texts of CGPs available

Paper copies were the most frequent format programs used to make texts of CPGs available to residents (54%). Five percent of the program directors reported using no specific strategy to make texts of CPGs available to the residents. Making texts of CPGs available through PDAs was associated with family medicine specialty (OR = 3.92; 1.94 to 7.93).

### Barriers

The top reported barrier was the time constraints on faculty (56%). Twenty two percent of responding program directors reported no barriers. Considering time constraints on faculty as a barrier was associated with 26 to 50% of residents being international medical graduates (OR = 2.05; 95% CI 1.15 to 3.66).

## Discussion

We conducted a survey of family medicine and internal medicine residency programs to determine how family medicine and internal medicine residency programs in the US teach the content of CPGs to their residents. Fourteen percent of program directors reported having written goals and objectives related to CPGs. The most frequently taught aspect of CPGs was the content of specific CPGs (76%). Educational activities were predominantly didactic in nature (76%). Auditing for adherence by residents was the primary evaluation strategy (44%), while 36% of program directors reported conducting no evaluation. Programs made texts of CPGs available to residents most frequently in the form of paper copies (54%), while the most important barrier was time constraints on faculty (56%).

This study has two main strengths. First, the survey questionnaire was rigorously designed, pretested, and is based on the EPOC review group typology of implementation strategies and ACGME definition of curriculum. Second, and to our knowledge, this is the first survey attempting to describe the teaching of the content of CPGs in both family medicine and internal medicine residency programs.

The main limitation of this study is the potential for selection bias with a response rate of 52%. This response rate, however, is consistent with the mean response rate of 54% to surveys of physicians published in medical journals [18]. On the other hand, surveys of pediatricians were found to be very consistent in showing only small amounts of response bias regardless of the response rate [20]. If selection bias existed, it is likely that teaching of the content of CPGs in the programs of responding directors would be of higher quality than in those of non-responding directors. This implies that our results could reflect an optimistic characterization of teaching of the content of CPGs in general, emphasizing the need for improvement. Another important limitation is that the study did not address important aspects of training in CPGs such as bedside teaching (*i.e.*, in concert with the care of individual patients) and integrating guidelines in medical decision making. These are however more challenging research questions that would require different study designs and different target populations to address them. In addition, the questionnaire was not detailed enough to capture details of the educational strategies (*e.g.*, type of auditing); however, this was intended to keep the questionnaire relatively short and increase response rate.

If teaching of the content of CPGs is considered an important part of the curriculum, programs will need to develop relevant goals and objectives. Goals and objectives offer a general focus, clarify expectations for learning and attainment, and relate them to residents' clinical and didactic activities [21]. Goals and objectives are also important for any evaluation process because outcomes are results providing evidence that goals and objectives have been accomplished [21]. We believe that such goals and objectives should cover basic skills, such as searching for and critically appraising CPGs, and more advanced skills, such as integrating CPGs in medical decision making, particularly those decisions that are sensitive to patients' values and preferences and for which guideline implementation is not equivalent to 100% adherence.

It is encouraging that the majority of programs teach the content of specific guidelines. The challenge remains however with the teaching strategy. The effectiveness of the top two strategies used by the majority of programs (*i.e.*, didactic sessions and journal clubs) is not supported by research evidence. A systematic review assessing the effectiveness of didactic sessions concluded that they are unlikely to change professional practice [22]. A review of the literature about the use of journal clubs identified no studies assessing their impact on adherence to CPGs [23].

On the other hand, the effectiveness of two teaching strategies used by a minority of programs (i.e., interactive sessions and audit and feedback) is supported by high quality research evidence. A systematic review assessing the effectiveness of interactive workshops concluded that they can result in moderately large changes [22]. Another systematic review found that audit and feedback can be effective in improving professional practice [24]. The effects are small to moderate, but likely to be greater when baseline adherence to recommended practice is low and when feedback is delivered more intensively [24]. Furthermore, the improved compliance may be generalizable to recommended practices not directly targeted for audit[25]

The time constraints on faculty being the most reported barrier to teaching of the content of CPGs, potential solutions include integrated approaches to evidence-based practice and teaching [26], resident-led small-group teaching [27], online tutorials [28], and educational games [29]. The effectiveness of some of these strategies remains to be proven. While the limited availability of CPGs was found in the past to be a barrier to the use of CPGs [30], the findings of this survey shows an improvement in guidelines accessibility in both paper or electronic forms, as also suggested by a recent survey of family medicine residency directors [31].

A number of reported characteristics of the teaching of CPGs were better for family medicine than for internal medicine residency programs (*e.g.*, identifying and locating CPGs) and vice versa (*e.g.*, conducting objective assessment of knowledge). However, the overall results are similar for the two specialties. The challenges are thus not unique to one of the two disciplines highlighting the need for collaborative efforts. We had hypothesized that a lower percentage of international medical graduates would be associated with better characteristics of teaching CPGs; although some of the associations were statistically significant, they were not consistent enough to support our hypothesis.

## Conclusion

The findings of this study have important implications for graduate medical education. Taking into account competing demands and requirements, program directors need to consider the value of teaching of the content of CPGs on the clinical care and educational levels (evidence-based medicine user model [32]). The teaching of the content of CPGs can benefit from adherence to the ACGME guidelines for curricular design. A particular attention should be given to the educational strategies used given their varying effectiveness.

The findings have also important implications for research. Time constraints on faculty being the major barriers, research should evaluate the effectiveness of innovative strategies that may consume less faculty time, such as online tutorials [28], case-based on-line learning [33], and educational games [29]. An additional challenge is to identify ways to facilitate incorporation of CPGs into decision making rather than simple conformance [8]. Future research should also investigate in other educational setting, the interesting association between the female gender and the higher number of years as program director with positive curricular characteristics. However, the major question to explore remains whether teaching of the content of CPGs in residency influences future practice behavior of physicians, particularly integrating CPGs in decision making in a manner that is consistent with the individual patient values and preferences.

## Competing interests

The authors declare that they have no competing interests.

## Authors' contributions

Authors contributions: conception and design: EAA, RM, MCW, AM, and HJS; acquisition of data: EAA, RM, AS, MCW, AM, and TR; analysis and interpretation of data: EAA, GHG, and HJS; drafting of manuscript: EAA; critical revision of the manuscript for important intellectual content: EAA, RM, AS, MCW, AM, TR, GHG, and HJS; obtaining of funding: EAA, RM, and HJS.

## Supplementary Material

Additional file 1 **Survey questionnaire**. Reproduces the questionnaire sent to the study participants. Click here for file
