# Supplementary material for: Curricula for teaching the content of clinical practice guidelines to family medicine and internal medicine residents in the US: a survey study
Source: Implement Sci. 2009 Sep 21;4:59. doi: 10.1186/1748-5908-4-59 (PMC2753632; doi:10.1186/1748-5908-4-59)
Supplement: Additional file 1 — Survey questionnaire. Reproduces the questionnaire sent to the study participants. [file 1748-5908-4-59-S1.DOC]

**Survey questionnaire**

1. My program has written goals and objectives for teaching Clinical Practice Guidelines (CPGs) as a topic (not only in the context of a specific disease)

 Yes

 No

1. My program teaches the following aspects of CPGs: (Check all that apply)

 Identifying and locating CPGs

 Critical appraisal of CPGs

 Content (*i.e.*, recommendations) of specific CPGs

 How to deal with conflicting CPGs

 Other (please specify) __________________________________________

 None of the above

1. Educational activity used to teach the content of specific CPGs: (Check all that apply)

 Making texts of CPGs available to residents

 Didactic sessions

 Interactive sessions

 Journal Club

 Audit and feedback to residents about their adherence to CPGs

 Self-audit by residents for adherence to CPGs

 Educational games (*e.g.*, Jeopardy! style game)

 Other (please specify): _________________________________________

 None of the above

1. Texts of CPGs are made available to residents through: (Check all that apply)

 The website of the program

 The server(s) of the affiliated hospital(s)

 email distribution

 PDA versions

 Paper copies

 Other: (please specify): __________________________________________

1. My program evaluates the teaching of CPGs through: (Check all that apply)

 Objective assessment of residents’ knowledge of content of specific CPGs

 Assessment of residents’ attitude towards CPGs

 Auditing of residents’ adherence to CPGs

 Assessment of residents’ satisfaction with CPGs teaching activities

 (please specify): __________________________________________

 None of the above

1. The major barriers to teach CPGs in my program: (Check all that apply)

 Limited access to CPGs

 Insufficient interest among residents

 Insufficient interest among faculty

 Time constraints on residents

 Time constraints on faculty

 Other: (please specify): ___________________________

 No barriers

1. US geographical region of residency program:

 Northeast

 South

 Midwest

 West

1. Residency program best described as *primarily*:

 Community based

 University based

 Military based

 Other:____

1. Total number of residents: ______
2. Percentage of residents who are international medical graduates:

 <25%

 25 to 50%

 51 to 75%

 >75%

1. Program director:

 Female

 Male

1. Number of years as program director: ______
